# A combination extract of *ginseng*, *epimedium*, *polygala*, and *tuber curcumae* increases synaptophysin expression in APPV717I transgenic mice

**DOI:** 10.1186/1749-8546-7-13

**Published:** 2012-06-09

**Authors:** Jing Shi, Jinzhou Tian, Xuekai Zhang, Mingqing Wei, Long Yin, Pengwen Wang, Yongyan Wang

**Affiliations:** 1 Department of Neurology, Dongzhimen Hospital, Beijing University of Chinese Medicine, Beijing 100700, China; 2 Clinical Neuroscience Research Group, The University of Manchester, Hope Hospital, Stott Lane, Salford, Manchester, M6 8HD, UK; 3 Key Laboratory of Chinese Internal Medicine (Beijing University of Chinese Medicine), Ministry of Education, Beijing, China; 4 Institute of Clinical Medicine, China Academy of Chinese Medical Sciences, Beijing, 100700, China

## Abstract

**Background:**

The density of presynaptic markers of synaptic communication and plasticity, especially synaptophysin (SYP), is significantly correlated with cognitive decline and the progression of Alzheimer’s disease (AD), indicating that synaptic protection is an important therapeutic strategy for AD. This study aims to investigate the synaptic protective effects of a combination of several active components extracted from the Chinese herbs *ginseng*, *epimedium*, *polygala* and *tuber curcumae* (GEPT), in the brains of APPV717I transgenic mice.

**Methods:**

Three-month-old APPV717I mice were arbitrarily divided into 10 groups (n = 12 per group): APP groups receiving vehicle treatment for four or eight months (model groups), three dose groups of GEPT-treated mice for each treatment period, and donepezil-treated mice for each treatment period. Three-month-old C57BL/6 J mice (n = 12) were also given vehicle for four or eight months (control groups). Vehicle, donepezil or GEPT were intragastrically administered. Immunohistochemistry (IHC) and Western blot analysis were used to assess protein expression in the hippocampal CA1 region and ratios of SYP to β-actin levels in hippocampal tissue homogenate, respectively.

**Results:**

Both IHC and Western blot revealed a decrease in SYP levels in the CA1 region of 7- and 11-month-old APPV717I transgenic mice compared with the control groups, whereas SYP levels were increased in donepezil- and GEPT-treated transgenic mice compared with the APP group. There was a significant difference in the levels of SYP detected by IHC between the GEPT high-dose group and the APP group after 4 months of treatment, and there were significant differences between all three GEPT groups and the APP group after 8 months of treatment. Western blotting showed that the SYP protein–β-actin ratio was decreased in APP mice, while donepezil- and GEPT-treated transgenic mice showed increased trends in the SYP protein–β-actin ratios.

**Conclusion:**

GEPT increases SYP expression and protects synapses before and after the formation of amyloid plaques in the brains of APPV717I transgenic mice.

## Background

Alzheimer’s disease (AD), the most common cause of dementia in the elderly, currently affects about 26 million patients worldwide [1]. It is characterized clinically by a progressive impairment of cognitive functions, such as learning and memory, and pathologically by the accumulation of β-amyloid peptides (Aβ), neurofibrillary tangles (NFTs) and neuronal degeneration [2]. Although Aβ accumulation in neurons plays an important role in AD pathogenesis [3,4], changes in the density of presynaptic markers, specifically synaptophysin (SYP), were suggested to be better correlated with disease progression and cognitive decline [5-9]. Moreover, evidence from transgenic mouse models with abundant Aβ deposition shows that deficits in synaptic communication and plasticity are crucial to the development of the disease. These synaptic changes usually occur before Aβ accumulation, and are strongly correlated with soluble nonfibrillary species of Aβ [10-12].

Previous studies have shown that a combination of herbal extracts called GEPT—consisting of extracts from *ginseng*, *epimedium*, *polygala*, and *tuber curcumae*[13]—can markedly enhance learning and memory in an AD rat model [14]. GEPT can also reduce the level of Aβ in APPV717I transgenic mice by inhibiting γ-secretase (or presenilin-1 (PS1)) and promoting insulin-degrading enzyme (IDE) and neprilysin (NEP) [13]. A 24-week Phase II clinical study [15] showed that GEPT significantly improved the cognitive function of patients with early-stage AD.

Synapses are considered to be the earliest site of AD pathology, and the rate of synaptic loss is directly related to the progression of the disease [16,17]. The pattern of synaptic protein-specific reductions throughout the brain reveals that synaptic loss is most commonly seen in the hippocampus, compared with other brain regions [18]. Among well studied synaptic proteins in dementia, SYP is the most abundant integral synaptic vesicle protein; its levels are often measured in attempts to quantify synapses [19]. Also, SYP is suggested to be involved in regulating assembly of soluble N-ethylmaleimide-sensitive fusion protein (NSF) attachment protein receptors (SNAREs), vesicle fusion, endocytosis and vesicle recycling [20-22]. SYP immunoreactivity has been reported to be absent from neurons containing the oligomeric form of Aβ, but it detected on those containing the fibrillar or monomeric forms of Aβ [23].

Neither cholinesterase inhibitors (*e.g.* donepezil) nor N-methyl-D-aspartate receptor antagonists (*e.g.* memantine), which are currently used to treated AD, are disease-modifying treatments that stop or slow disease progression [24]. Although there is evidence supporting the use of single herbs or herbal formulations to complement approved medicines, current evidence supporting their use alone is inconclusive [24]. Therefore, this study aims to investigate the potential effects of combination of herbs in treating AD.

## Materials and methods

### Preparation of drugs

GEPT (called GETO in our previous publications), consists of 35% ginsenoside extracted from *ginseng*, 35% flavonoid glycoside extracted from *epimedium*, 15% tenuifolin extracted from *polygala*, and 15% curcumin from *tuber curcumae*. GEPT was provided by Henan Wanxi Pharmaceutical Company Limited (batch no. 20010923, China). Hydrochloric acid donepezil tablets were provided by Eisai Pharmaceutical Company Limited (batch no. 090508A, China). GEPT was dissolved in 0.5% carboxymethyl cellulose (CMC) (Sigma, USA) at a concentration of 30 mg/mL, and donepezil tablets were crushed and dissolved in 0.5% CMC at a concentration of 0.092 mg/mL.

### Animals and medicine administration

Three-month-old APPV717I mice and C57BL/6 J mice (non-transgenic inbred mice, used as vehicle controls) were purchased from the Institute of Experimental Animals, Chinese Academy of Medical Sciences & Peking Union Medical College (Beijing, China). The APP/V717I transgenic mice had a C57BL/6 J genetic background and carried mutated human APP-CT100 containing the London mutation, V717I, which results in increased generation of Aβ_42_ and AD-like pathological changes [25]. All animals were housed in the Pharmacological Experiment Center of Dongzhimen Hospital, Beijing University of Chinese Medicine, Beijing, China. They were maintained in a temperature-controlled (24°C) pathogen-free vivarium, on a 12:12-h light:dark cycle (12-h light [06:00 to 18:00], 12-h dark [18:00 to 06:00]) with free access to food and water. All experimental procedures were performed in compliance with the Provision and General Recommendations of the National Institutes of Health Guide for the Care and Use of Laboratory Animals and were approved by the Animal Research Ethics Board of Beijing University of Chinese Medicine.

APPV717I transgenic mice were arbitrarily divided into 10 groups (n = 12 per group) and received intragastrically administrated vehicle or medicines. APP groups were given 0.5% CMC and donepezil groups were given donepezil (APP + D; 0.92 mg/kg/d) for 4 or 8 months. GEPT was administered at three dosage levels, with a separate group for each: low dose (APP + Gl; 0.075 g/kg/d), medium dose (APP + Gm; 0.15 g/kg/d), and high dose (APP + Gh; 0.30 g/kg/d), also for 4 or 8 months. Male C57BL/6 J mice served as vehicle controls (n = 12) and were given 0.5% CMC for 4 or 8 months.

### Behavioral assessments

Spatial learning and memory were assessed in orientation navigation tests using the Morris water maze (MWM) as described previously [13]. After 4 or 8 months of treatment, all mice underwent testing in the MWM, which consisted of two days of learning and memory training, followed by three days of probe trials. The animals’ swim paths and the numbers of annulus crossings were recorded on videotape; the percentage of time spent in each quadrant and the average swim speed were determined from these videotapes.

### Tissue preparation

All behaviorally tested mice were deeply anesthetized with 10% chloral hydrate (Loogene Biotechnology Co., Ltd, China) (40 mg/kg, i.p.) and pericardially perfused with heparinized 0.9% saline, prior to removal of the brain. Brains were immersion-fixed in 4% paraformaldehyde (Sun Biomedical Technology Co., Ltd, China) overnight at 4°C and then processed in a phosphate-buffered saline (PBS) solution containing 30% sucrose. Seven days later, brains were embedded in paraffin. Serial coronal sections of the hippocampus were cut at 35-μm intervals for immunohistochemistry (IHC) staining. The brains of three arbitrarily selected mice were separated according to regions and snap frozen for Western blot.

### IHC staining

Brain sections were deparaffinized and rehydrated in distilled water. Antigens were then unmasked in 0.01 M citrate buffer (Sun Biomedical Technology Co., Ltd, China) by microwave, and endogenous peroxidase activity was quenched by 0.3% hydrogen peroxide (Sun Biomedical Technology Co., Ltd, China) in methanol (Sinopharm Chemical Reagent Co., Ltd, China) for 20 min at room temperature. The sections were then blocked with 3% bovine serum albumin (Sigma-Aldrich Co. LLC, USA) in PBS for 30 min at 37°C. After excess serum was removed, sections were incubated with primary antibody against SYP (1:400; ab23754, Abcam, USA) in humidified boxes at 4°C overnight. They were then washed again and incubated with biotin-conjugated secondary antibodies (1:300, Fuzhou Maixin Ltd, China) for 30 min at 37°C, then washed again and incubated with Streptavidin-Biotin Complex (SABC) (Wuhan Boster Bioengineering Co., Ltd, China) for 1 h at 37°C. Sections were subsequently developed using the chromogen 3’,3-diaminobenzidine tetrachloride (DAB), after which they were dehydrated and coverslipped. All brain sections chosen for staining were on a similar sagittal plane and contained approximately the same area of hippocampus. SYP average optical density (AOD) was measured in immunostained sections, following the instructions of the Image Pro Plus 6.0 software (Media CY Company, USA). “Nonspecific” IHC staining in sections was chosen as the control area for comparison with the SYP-immunopositive area in the neurons of the dentate gyrus.

### Western blot

Western blots were performed based on a previously described method [13]. Briefly, snap-frozen brain tissues from hippocampus and cortex were weighed and homogenized in brain tissue lysis buffer (Loogene Biotechnology Co., Ltd, China) using a small pestle on ice, at a ratio of 1:10 (w/v) for 2 min, and incubated on ice for 30 min. Homogenates were centrifuged at 4300 x *g* at 4°C for 30 min, and supernatants were collected. The level of protein in the supernatants was determined by the modified Bradford method [26] using Coomassie Brilliant Blue G-250 (Nanjing Jiancheng Bioengineering Institute, China). Loading buffer was added to samples at a ratio of 4:1, after which samples were placed in boiling water for 5 min and then immediately chilled on ice. Aliquots (10 μL) of each sample and 5 μL of marker (10–170 kDa) were loaded onto 10% acrylamide gels (Sigma-Aldrich Co. LLC, USA) and subjected to SDS-PAGE using the Bio-Rad mini gel system (Bio-Rad, USA). Proteins were then electro-blotted onto polyvinylidine difluoride membranes. Membranes were blocked with 5% milk at 4°C overnight, and then incubated with primary antibody (anti-SYP antibody, ab23754, 1:5000). After three washes with PBS containing 0.5% Tween 20 (PBST) (Sun Biomedical Technology Co., Ltd, China), membranes were incubated at room temperature for 1 h with anti-rabbit IgG (H&L) (Equitech Bio, Inc. USA) horseradish peroxidase-conjugated secondary antibody (Promega Co., USA) at 1:10,000 on a shaker. After three washes with PBST, blots were developed using Luminol reagent (Pierce Biotechnology, USA). Densitometric analysis of the blots was completed using Phoretix 1D software (Total Lab Ltd, UK). Expression of SYP protein is shown as the SYP protein-β actin ratio.

### Statistical analysis

All data were analyzed using SPSS 13.0 software (IBM Software, USA) and are presented as mean ± standard deviation (SD). One-way ANOVA with Tukey’s *post-hoc* test was used when comparisons were made between two groups. *P* < 0.05 was considered statistically significant.

## Results

### Spatial learning and memory abilities

During the 5-day spatial learning and memory test, training was done for the first two days and testing was done on the following three days. The average escape latencies of 7-month-old mice were all gradually decreased during the three day test. However, the average escape latencies in 7-month-old APPV717I transgenic mice (44.87 ± 11.98 s) were significantly longer than those in vehicle control mice of the same age (27.53 ± 14.21 s) (*P* = 0.006) on the fifth training day. The mice in treated APP groups showed decreased escape latencies in a two-minute test on the fifth training day. Of these, APP + D mice had the shortest escape latencies 30.58 ± 15.42 s (*P* = 0.047), followed by APP + Gh mice with a latency of (30.91 ± 13.01 s) (*P* = 0.036), APP + Gm mice with a latency of 31.42 ± 14.80 s (*P* = 0.049), and APP + Gl mice with a latency of 35.00 ± 15.75 s (*P* = 0.008). Similar results were found in 11-month-old mice (Tables 1 and 2).

**Table 1:** Escape latencies of 7-month-old mice

**Group**	**Day 3**	**Day 4**	**Day 5**
Control	36.80 ± 12.60	32.87 ± 15.5	27.53 ± 14.21
APP	50.33 ± 9.39^▲▲^	47.73 ± 8.65^▲▲^	44.87 ± 11.98^▲▲^
APP + D	38.25 ± 14.41^●^	34.42 ± 12.84^●^	30.58 ± 15.42^●^
APP + Gl	40.93 ± 14.11	36.93 ± 13.78^●^	35.00 ± 15.75
APP + Gm	42.92 ± 13.05	38.58 ± 14.15	31.42 ± 14.80^●^
APP + Gh	38.45 ± 14.12^●^	35.27 ± 14.01^●^	30.91 ± 13.01^●^

The effects of GEPT on escape latency in 7-month-old mice (n = 12) were assessed in orientation navigation tests using the Morris Water Maze (MWM). Data are expressed as mean ± standard deviation (SD)**. Control:** C57BL/6 J mice; **APP**: APPV717I mice; **APP + D**: APP mice treated with donepezil; **APP + Gl**: APP mice treated with GEPT (low dose); **APP + Gm**: APP mice treated with GEPT (medium dose); **APP + Gh**: APP mice treated with GEPT (high dose). *P* < 0.05 *vs.* APP mice alone; ^▴▴^*P* < 0.01 *vs.* control mice, one-way ANOVA.

**Table 2:** Escape latencies of 11-month-old mice

**Group**	**Day 3**	**Day 4**	**Day 5**
Control	39.17 ± 12.68	36.75 ± 14.72	33.08 ± 14.49
APP	51.33 ± 7.80^▲^	49.83 ± 7.78^▲^	47.17 ± 10.19^▲^
APP + D	42.25 ± 14.23	40.00 ± 12.69	34.83 ± 13.60^●^
APP + Gl	43.27 ± 10.32	37.64 ± 13.52^●^	37.55 ± 13.92
APP + Gm	41.60 ± 10.51	43.60 ± 11.61	35.50 ± 13.91^●^
APP + Gh	40.00 ± 12.34^●^	38.83 ± 14.45^●^	34.42 ± 12.38^●^

The effects of GEPT on the escape latency in 7-month-old mice (n = 12) were assessed in orientation navigation tests using the Morris Water Maze (MWM). Data are expressed as mean ± standard deviation (SD). **Control:** C57BL/6 J mice; **APP**: APPV717I mice; **APP + D**: APP mice treated with donepezil; **APP + Gl**: APP mice treated with GEPT (low dose); **APP + Gm**: APP mice treated with GEPT (medium dose); **APP + Gh**: APP mice treated with GEPT (high dose). *P* < 0.05 *vs.* APP mice alone; ^▴^*P* < 0.05 *vs.* control mice, one-way ANOVA.

### SYP expression levels

#### IHC staining

**Figure 1:**
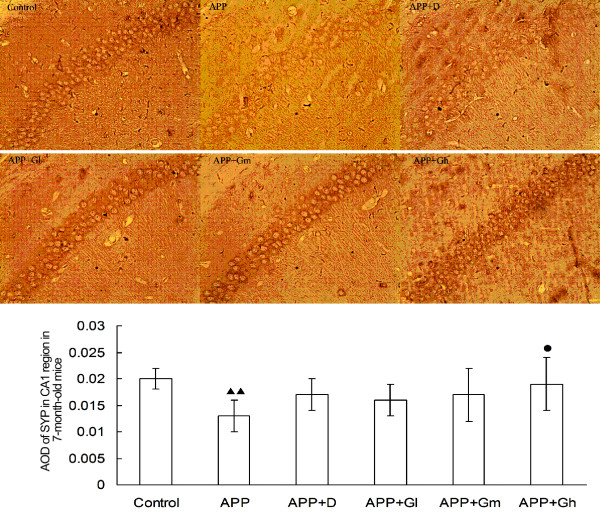
**Expression of SYP in the hippocampal CA1 region in 7-month-old mice.** The effects of GEPT on the expression of SYP in the hippocampal CA1 region in 7-month-old experimental mice were assessed by IHC staining. Average optical densities of the SYP positive neuronal area (anti-body for SYP, 1:400) are expressed as mean ± SD (n = 5). **Control**: C57BL/6 J mice; **APP**: APPV717I mice; **APP** + **D**: APP mice treated with donepezil; **APP** + **Gl**: APP mice treated with GEPT (low dose); **APP** + **Gm**: APP mice treated with GEPT (medium dose); **APP** + **Gh**: APP mice treated with GEPT (high dose). *P* < 0.05 *vs.* APP mice alone, *P* < 0.01 *vs.* APP mice; ^▴▴^*P* < 0.01 *vs.* control mice, ANOVA.

**Figure 2:**
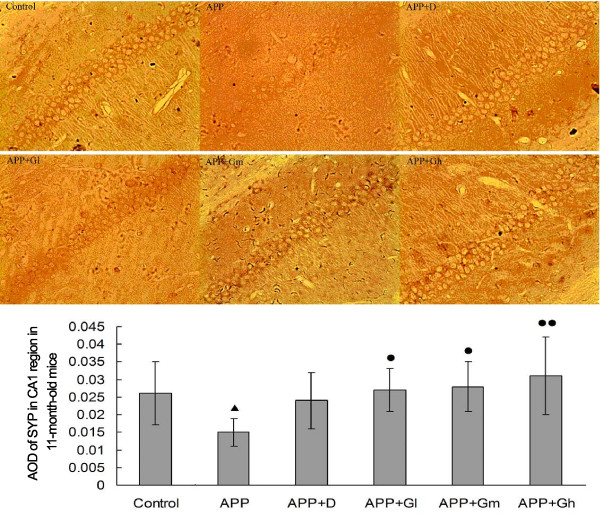
**Expression of SYP in the hippocampal CA1 region in 11-month-old mice.** The effects of GEPT on the expression of SYP in the hippocampal CA1 region in 11-month-old experimental mice were assessed by IHC staining. Average optical densities of the SYP positive neuronal area (anti-body for SYP, 1:400) are expressed as mean ± SD (n = 5). **Control:** C57BL/6 J mice; **APP**: APPV717I mice; **APP** + **D**: APP mice treated with donepezil; **APP** + **Gl**: APP mice treated with GEPT (low dose); **APP** + **Gm**: APP mice treated with GEPT (medium dose); **APP** + **Gh**: APP mice treated with GEPT (high dose). ^▴^*P* <0.05 *vs.* control group, *P* < 0.05 *vs.* APP group, *P* < 0.01 *vs.* APP group, one-way ANOVA.

IHC analysis showed a significant decrease in SYP expression through the measurement of AOD in the CA1 region of 7-month-old APPV717I transgenic mice (*P* = 0.002 compared with controls), while SYP expression was increased in donepezil- and GEPT-treated groups (Table 3). There was only a significant difference between the APP-Gh group and the non-treated APP group (*P* = 0.031). Similar results were obtained for SYP expression in the CA1 region of 11-month-old experimental mice, but at that age, there were also significant differences between the APP-Gm group and the APP group (*P* = 0.019), and between the APP-Gl group and the APP group (*P* = 0.032) (Table 3). There was no significant difference between the donepezil-treated group and the APP group in either 7- or 11-month-old experimental mice (Figures 1 and 2).

**Table 3:** Expression of SYP in the hippocampal CA1 region in 7- and 11-month-old mice

**Group**	**Average optical density**	
	**7-month-old mice**	**11-month-old mice**
Control	0.020 ± 0.002	0.026 ± 0.009
APP	0.013 ± 0.003^▲▲^	0.015 ± 0.004^▲^
APP + D	0.017 ± 0.003	0.024 ± 0.008
APP + Gl	0.016 ± 0.003	0.027 ± 0.006^●^
APP + Gm	0.017 ± 0.005	0.028 ± 0.007^●^
APP + Gh	0.019 ± 0.005	0.031 ± 0.011^●●^

The effects of GEPT extract on the expression of SYP in the hippocampal CA1 region in 7- and 11-month-old mice (n = 5 per group) were assessed by IHC staining. Average optical densities of the SYP positive neuronal area (anti-body for SYP, 1:400) are expressed as mean ± standard deviation (SD)**. Control:** C57BL/6 J mice; **APP**: APPV717I mice; **APP + D**: APP mice treated with donepezil; **APP + Gl**: APP mice treated with GEPT (low dose); **APP + Gm**: APP mice treated with GEPT (medium dose); **APP + Gh**: APP mice treated with GEPT (high dose). *P* < 0.05 *vs.* APP mice alone, *P* < 0.01 *vs.* APP mice; ^▴▴^*P* < 0.01 *vs.* control mice, one-way ANOVA.

### Western blot

**Figure 3:**
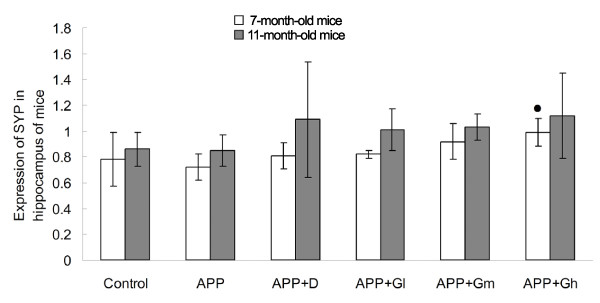
**Expression of SYP proteins (SYP–β-actin ratio) in hippocampal tissue homogenates of 7- and 11-month old experimental mice.** The effects of GEPT on the expression of SYP in hippocampal tissue homogenates of 7- and 11-month old experimental mice were determined by Western blot (anti-body for SYP, 1:5000) and subsequently analyzed by densitometry. β-actin served as an internal control. **Control**: C57BL/6 J mice; **APP**: APPV717I mice; **APP** + **D**: APP mice treated with donepezil; **APP** + **Gl**: APP mice treated with GEPT (low dose); **APP** + **Gm**: APP mice treated with GEPT (medium dose); **APP** + **Gh**: APP mice treated with GEPT (high dose). *P* < 0.05 *vs.* APP group, one-way ANOVA.

In 7-month-old experimental mice, Western blot analysis revealed a decrease in the ratio of SYP protein to β-actin (internal control) in the hippocampus of APP mice when compared to control mice, but there was no significant difference. However, SYP expression was increased in all donepezil- or GEPT-treated transgenic mice when compare to APP mice, and there was a significant difference in the levels of SYP between the APP-Gh group and the APP group (*P* = 0.041). Similar results were obtained for SYP expression in the hippocampus of 11-month-old experimental mice, while there was no significant difference between each group (Figure 3).

## Discussion

Although synaptic decline has been clearly shown to be related to the duration and severity of dementia in the AD process [8,16,17], and synapse loss has been shown to be positively correlated with neurons containing NFTs [27], the association between presynaptic proteins and senile plaques is not clear [18]. Meanwhile, only the soluble form of Aβ has been found to be a predictor of synaptic change in the entorhinal cortex and superior frontal gyrus [28]. SYP immunoreactivity has been reported to be absent around neurons containing the oligomeric form of Aβ, but it is present around those containing the fibrillar or monomeric forms [23]. Therefore, this study focused on the synaptic decline in APP/V717I transgenic mice and the protective effects of GEPT in 7- and 11-month old transgenic mice.

In APPV717I transgenic mice, the formation of amyloid plaques begins at around nine months of age [29,30], but learning and memory deficits have been detected as early as four months of age, and gradually become worse during the following 12 months; after 16 months of age the learning and memory deficits still exist, but are significantly less severe [31]. These findings indicate the critical involvement of amyloid peptides in defective LTP in APP transgenic mice. The escape latency in the MWM was prolonged, and the discrimination index was decreased in an object recognition test. To observe the synaptic protection effects of GEPT before and after amyloid plaque formation, 3-month-old APPV717I transgenic mice were treated with GEPT up to the ages of 7 and 11 months.

In previous studies, GEPT markedly enhanced learning function and memory abilities in AD rat models and significantly improved spatial learning and memory abilities in APPV717I transgenic mice following 8 months and 3 months of treatment with GEPT, suggests that GEPT could significantly prevent memory and cognitive impairments and delay memory decline in those dementia models [13,14]. The potential mechanism of GEPT action in the brains of APPV717I transgenic mice may be through the inhibition of PS1 activity rather than through inhibition of Beta-secretase enzyme (BACE1) and the promotion of IDE and NEP activity [13]. However, it is not known if GEPT has synaptic protective effects in APPV717I mice. In the present study, both IHC and Western blot analyses revealed a significant decrease in SYP levels in the CA1 region of the brains of 7- and 11-month-old APPV717I transgenic mice, whereas SYP levels were increased in donepezil- and GEPT-treated transgenic mice. There was a significant difference in SYP levels between the APP-Gh group and untreated transgenic mice aged 7 months. IHC analysis showed significant differences in SYP levels between each of the three GEPT-treated groups and untreated transgenic mice aged 11 months, whereas there were no significant differences between the donepezil-treated group and the nontreated transgenic mice group at the same age. Western blot analysis showed that the SYP protein–β-actin ratio was only decreased in APP mice when compared to vehicle-treated control mice, while the ratio was increased in donepezil- and GEPT-treated transgenic mice; however, there was no significant difference in ratios among each group. These data indicated that GEPT, especially at a high dose, can act as a synaptic protective agent in APP mice, before the formation of amyloid plaques. The present findings were consistent with those of a 24-week preliminary study of GEPT that showed a significant improvement in cognitive function in patients in the early stage of AD [15].

SYP, a well-studied synaptic protein in cases of dementia, is the most abundant integral synaptic vesicle protein and its levels are often measured in attempts to quantify synapses [19]. The present study investigated the influence of GEPT on SYP expression before and after the formation of amyloid plaques in the brains of APPV717I transgenic mice. The results not only revealed the mechanism of action of GEPT in providing synaptic protection, but also reveal its potential for the treatment of AD. However, there are still some outstanding issues in this study. First, SYP levels in the brains of APPV717I transgenic mice did not decrease between 7 and 11 months of age. This may be because a four-month period is not long enough for APP/V717I transgenic mice to show a clear decrease in SYP expression. However, our findings clearly showed that GEPT can time-dependently increase expression of SYP. Second, SYP expression in the brain is only one of a number of synaptic markers that are significantly correlated with disease progression and cognitive decline [5-9]. Therefore, further studies in other AD transgenic mice, over a longer time span or investigating other synaptic markers, should be conducted.

## Conclusion

GEPT increases SYP expression and protects synapses before and after the formation of amyloid plaques in the brains of APPV717I transgenic mice.

## Abbreviations

SYP: Synaptophysin; GEPT: *Ginseng*, *epimedium*, *polygala* and *tuber curcumae*; IHC: Immunohistochemistry; Aβ: β-amyloid peptides; NFTs: Neurofibrillary tangles; PS1: γ-secretase (or presenilin-1); IDE: Insulin-degrading enzyme; NEP: Neprilysin; SNAREs: N-ethylmaleimide-sensitive fusion protein (NSF) attachment protein receptors; CMC: Carboxymethyl cellulose; MWM: Morris water maze; PBS: Phosphate-buffered saline; SABC: Streptavidin-Biotin Complex; DAB: Chromogen 3’,3-diaminobenzidine tetrachloride; AOD: Average optical density; PBST: PBS containing 0.5% Tween 20; BACE1: Beta-secretase enzyme.

## Competing interests

The authors declare that they have no competing interests.

## Authors’ contributions

JT and JS designed the study, conceived, wrote and finalized the manuscript. LY conducted the experiments. MW and XZ analyzed and prepared the manuscript. PW conducted the Western blot and IHC experiments. YW reviewed the design of the study and was involved in discussions of the study. All authors read and approved the final manuscript.
